# Male convict cichlid 11-ketotestosterone levels throughout the reproductive cycle: an exploratory profile study in laboratory and field populations

**DOI:** 10.7717/peerj.949

**Published:** 2015-05-07

**Authors:** Natalie April van Breukelen, Jennifer L. Snekser, Murray Itzkowitz

**Affiliations:** 1 La Paz Community School, Playa Flamingo, Guanacaste, Costa Rica; 2 LIU Post, Department of Biology, Brookville, NY, USA; 3 Lehigh University, Department of Biological Sciences, Bethlehem, PA, USA

**Keywords:** Convict cichlid, 11-ketotestosterone, Costa Rica, 11KT, Biparental

## Abstract

The convict cichlid (*Amatitlania nigrofasciata*) has been extensively examined in relation to many behavioral topics, such as courtship, pair-bonding, bi-parental care, and territoriality. Recently, this model species has been utilized in studies on genetics, endocrinology, and neuroanatomy, with an ultimate goal of connecting behavior with its underlying mechanisms. The goal of this study was two-fold: (1) profile the circulating levels of plasma 11KT in the male convict cichlid at multiple points during the reproductive cycle and (2) generally compare the hormonal profiles of the widely used laboratory populations and those of a free-living population in the streams of Costa Rica. The results of the field experiment showed that male convict cichlids had higher levels of circulating 11KT during courtship and lower during the parental care and non-breeding phases. The profile of the laboratory population was similar to the profile of the free-living individuals, with significantly higher levels of 11KT occurring during courtship than during parental care, though the level of 11KT during non-breeding phase was elevated in the laboratory. The high levels of 11KT during courtship and low levels of 11KT during parental care found in both the field and the laboratory is similar to what has been reported in other species of teleosts, and may suggest an important function of 11KT in the expression of courtship behavior and the subsequent onset of parental behaviors in this model species.

## Introduction

The examination of hormone levels in a variety of animals have shown strong correlations between the expression of different behaviors and fluctuations in specific hormones, providing us with a better understanding of the probable proximate causes of behavioral expression. The majority of behavioral neuroendocrinological experiments on vertebrates have been performed within the laboratory setting, likely due to the obvious advantages of being able to control for variables and the ease of assay-work at the bench top (Calisi & Bentley, 2009). Yet, evidence has shown that stark differences can be seen between laboratory and wild-populations in terms of both their behavior and measured hormone-levels (e.g., Cardwell et al., 1996; Woodruff, Lacey & Bentley, 2010; Dickens & Bentley, 2014; and those reviewed by Calisi & Bentley, 2009). In a comparison of wild and captive male cowbirds (*Molothrus ater*), for example, substantial differences were seen in the levels of circulating testosterone. Wild paired males had higher concentrations of circulating testosterone than unpaired males early in the season, with testosterone peaking later in the season during social hierarchy formation and mate acquisition (Dufty & Wingfield, 1986a). Captive paired males had a more rapid increase and longer maintenance of high testosterone levels throughout the season (Dufty & Wingfield, 1986b). Studies such as these provide evidence that laboratory conditions have the potential to influence the underlying physiology of animals.

Here, we seek to explore the circulating hormone levels of both laboratory-housed and wild field populations of a biparental teleost, the convict cichlid (*Amatitlania nigrofasciata* (alternatively *Amatitlania siquia*, *Archocentrus nigrofasciatus*, and *Cichlasoma nigrofasciatum*)). In general, studies of the behavioral endocrinology of fishes have been particularly interested in the hormone 11-ketotestosterone (11KT), which is the predominant androgen in many teleost species. This non-aromatizable androgen (i.e., it does not convert to estrogen) (Borg, 1994; Liley & Stacey, 1983) is responsible for the development of sex organs and sexual characteristics, as well as sexual maturation in many teleosts examined (reviewed by Borg, 1994). The circulating levels of 11KT change throughout the reproductive cycle and several species of teleosts have similar reproductive profiles, with the highest levels occurring prior to spawning (presumably during courtship) followed by a decrease with the onset parental care (Sikkel, 1993; Knapp, Wingfield & Bass, 1999; Gazola & Borella, 1997; Ros et al., 2003; Barnett & Pankhurst, 1994; Pall, Mayer & Borg, 2002a; Pankhurst & Peter, 2002) (though the opposite trend is seen in the blue-banded goby (Rodgers, Earley & Grober, 2006)). It has been suggested that courtship behavior of male teleosts is likely affected by 11KT, as castration-induced declines in courtship were most effectively reinstated by treatment with 11KT or an androgenic precursor, 11-ketoandrostendione (11KA), (Borg, 1987; Salek, Sullivan & Godwin, 2001; Pall, Mayer & Borg, 2002b). Additionally, as the levels of 11KT decline over the breeding cycle, courtship behavior also declines (Pall, Mayer & Borg, 2002a). This profile may indicate a functional role for 11KT, and its fluctuation, in the expression of all aspects of reproductive behavior, from courtship through parental care.

Our study intentionally focused on the convict cichlid because it has become a model species that is widely used for behavioral studies and is of increasing interest to researchers that seek to connect behavior with genetics (e.g., Lee-Jenkins et al., 2011), neuroanatomy (e.g., O’Connell, Matthews & Hofmann, 2012; Nesjan et al., 2014), and endocrinology (e.g., O’Connell, Matthews & Hofmann, 2012; Oldfield & Hofmann, 2011; Sessa, Harris & Hofmann, 2013; van Breukelen, 2013). These freshwater cichlids occur throughout Central America in rivers, streams and lakes (Bussing, 1998). In addition to the multitude of laboratory experiments, the convict cichlid also lends itself to easy study within the field, in both Costa Rica (e.g., Wisenden, 1994; Wisenden, 1995; Wisenden et al., 2008; Lee-Jenkins et al., 2011; Snekser et al., 2011; van Breukelen, 2015) and Nicaragua (e.g., McKaye, 1986; Alonzo, McKaye & Van den Berghe, 2001; Lehtonen, 2008; Wisenden et al., 2008), further adding to their value as a model species. The many decades of behavioral research on this species has provided an extensive framework for integrative studies that will reveal more about the connections between behavior and its underlying mechanisms.

Behaviorally, the convict cichlid has been the interest of many researchers for many decades because of their complex social system. After reaching sexual maturity, males and females court each other in order to form exclusive pair bonds (Keenleyside, 1991; Wisenden, 1994). The pair-bonded individuals guard a breeding cave together, where they spawn a single brood and protect their eggs for up to four days. Following hatching, the young experience an additional four days during which they are absorbing their yolk sacs and are unable to swim, a stage traditionally referred to as the “wriggler” stage (Noakes, 1991). During this wriggler stage, the young stay within the cave and both parents provide protection (Wisenden, 1995). When they are able to swim, the fry leave the cave and stay with their parents for up to six weeks. Both parents contribute to the young’s protection from predators and provide them with food (detritus) via leaf-flipping (Wisenden, Lanfranconi-Izawa & Keenleyside, 1995), though there seem to be distinct sex-typical roles, with females spending more time near young and males defending more against predators (Wisenden, 1994). Following the maturation of the fry, the parents no longer stay within their pair bond, and if they mate again during the breeding season (or the next) it is most typically with a new mate (Wisenden, 1995). Thus, these serially monogamous, biparental fishes provide a very interesting model system to study.

In regards to the convict cichlid and hormones specifically, recent studies have suggested a functional role for 11KT in the exhibition of social behaviors. In a laboratory study, O’Connell, Matthews & Hofmann (2012) found that 11-KT was higher in solitary male convict cichlids (who spawned and subsequently had their brood and mate removed) compared to biparental males and single fathers. Additionally, in the laboratory, male convict cichlids in “social” situations (with a male competitor or with a gravid female) had levels of 11KT that were overall high than when in “nonsocial” (solitary) situations (Sessa, Harris & Hofmann, 2013). Blocking 11KT (and all androgens) with flutamide (an androgen receptor blocker) inhibited courtship behavior, but not aggression in male convict cichlids, in both laboratory and field populations (van Breukelen, 2013). As with the previously studied teleost species, it seems as though 11KT has a definitive role in the expression of convict cichlid behavior, specifically those related to courtship.

This study had two goals: (1) profile the circulating levels of plasma 11KT in the male convict cichlid at multiple points during their natural reproductive cycle and (2) generally compare those hormonal profiles of the widely used laboratory populations and those of a free-living population in the streams of Costa Rica. Although O’Connell, Matthews & Hofmann (2012) and Sessa, Harris & Hofmann (2013) have examined 11KT levels in convict cichlids previously, we sought to examine hormonal levels within additional social situations, specifically during non-breeding, social interactions, as well as during courtship in the early stages of pair bonding and actively parenting. We expected differences across the reproductive cycle, as shown previously. In contrast to the previous studies, our second aim was to compare our laboratory findings to the hormone levels of convict cichlids in their natural habitat. We anticipated similar hormone profiles for the laboratory and Costa Rican populations. Achieving our specific goals will both add to our knowledge of the general hormonal profiles of various teleosts, as well as provide evidence of the similarity and/or differences of wild and captive populations of these extensively studied fish.

## Materials and Methods

### Field

The field portion of this study was conducted in the Rio Cabuyo, located in the Lomas Barbudal Biological Reserve (Guanacaste, Costa Rica) during the dry season from December 20, 2007 to January 5, 2008. All research was conducted with permission from Ministerio del Ambiente y Energia (MINAE permiso #48). Males were observed and were classified as either non-breeding, courting, or parental based upon their exhibited behavior. Adult males observed congregating in large mixed sex groups with no overt courtship or aggressive behavior were classified as non-breeding. Males observed expressing courtship behavior towards a female and aggression towards males were classified as courting. Males observed interacting with a mate and caring for offspring were classified as parental.

After observations to determine reproductive stage, males were immediately caught using a weighted drop net of transparent monofilament line and a hand net. The captured male was placed on a wet, flat, floating piece of plastic. Blood was drawn from the caudal vein using a heparinized syringe with a 26-gauge needle. The syringe was inserted in the caudal peduncle, 2–3 scale rows below the midline of the body. The needle was inserted until it met resistance and then slight back pressure applied. This type of blood draw method has been used successfully in other species (Pankhurst, 1990). Approximately 0.04–0.08 ml were drawn per subject and placed on ice. All blood draws occurred within 5 min of capture. Two scales on the caudal peduncle were removed to facilitate the blood draw and to identify males that had been sampled. After sampling, males were given several minutes to recover and released. Sampled males were between 60 mm and 80 mm and were only sampled once. Blood was kept on ice and transported to the field station and centrifuged at 2,000 × g for 10 min. The resultant plasma was frozen and transported back to Lehigh University for assaying.

### Laboratory

The laboratory portion of this investigation was conducted at Lehigh University in accordance with IACUC approval (protocol approval ID #15). Laboratory subjects were either directly purchased from a fish distributor (Seven Star Tropical, Philadelphia PA) or descended from purchased fish. The fish used were adults and were not sexually naive. Prior to use, the convict cichlids were maintained in uni-sexual groups of approximately 20–30 individuals, in 475 L stock tanks. The laboratory was maintained at 27 °C, with a light:dark cycle of 14:10 h. Fish were fed commercial fish food.

Adult male convict cichlids were assigned to one of 3 groups; non-breeding, courting, and parental. Males in the non-breeding group were housed in male-only groups for at least 2 months in 475 L stock tanks. For the experiment, males were caught from the stock tank and immediately sampled. Males in the courting group were placed with a female and another male into a 300 L tank with an artificial breeding site for 48 h. Only males that were observed to be actively engaged in courtship behavior towards the female were sampled. Males in the parental group were males that had spawned with a female and were housed in a 300 L tank with mate and offspring either in the larval stage or free swimming fry. Only males observed to be actively engaged in parental care behavior were sampled. After sampling, males from all groups were placed in a separate tank to ensure that each male was only sampled once. Each male was caught and blood was drawn as described above. The blood samples were immediately centrifuged at 2000 × g for 10 min and the resultant plasma stored at −80 °C until assayed.

### 11-Ketotestosterone assay

Samples were analyzed using a commercially available enzyme immunoassay (EIA) kit (Cayman Chemical; Ann Arbor, Michigan, USA) following the protocols provided. The EIA kits have been used with other species of teleosts (Chu-Koo et al., 2009; Earley & Hsu, 2008; Lorenzi et al., 2008; Kidd, Kidd & Hofmann, 2010) and with convict cichlids (O’Connell, Matthews & Hofmann, 2012; Sessa, Harris & Hofmann, 2013) and are reported to have a specificity of 100% for 11KT and less than 0.01% for T and other androgens, with low cross-reactivity. The laboratory and field blood samples were analyzed separately, each using two 96 well plate 11KT EIA kits. All samples were run in duplicate and at a 1:100 dilution. For the laboratory assays, the intra-assay variations were calculated to be 6.45% and 5.15% and the inter-assay variation was 14.7%. The intra-assay variations for the field assays were 6.32% and 5.56% and the inter-assay variation was 6.03%.

### Statistical analysis

Because the behavioral groups sampled in the field and in the laboratory were similar, but not identical, we analyzed the field and laboratory samples separately, using an ANOVA (SPSS 15.1) followed by Tukey HSD post-hoc comparison to determine differences in the plasma levels of 11KT among the 3 groups.

## Results

### Field

**Figure 1:**
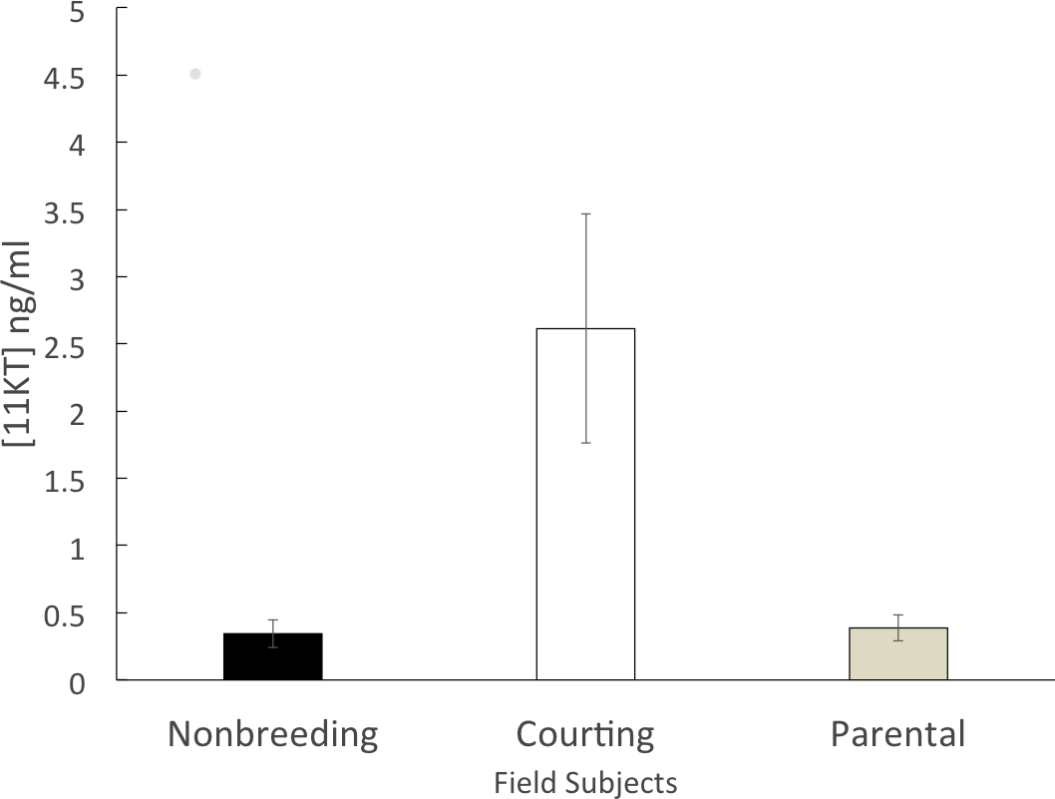
Mean (± SEM) concentration of plasma 11KT of male convict cichlids in a Costa Rican stream. Non-breeding males (black) and parental males (gray) were significantly different from courting males (white).

The non-breeding group (*n* = 6) had a mean circulating 11KT level of 0.344 ± 0.102 ng/ml. The parental group (*n* = 5) expressed a similar level of circulating 11KT (0.387 ± 0.097 ng/ml). The courting group (*n* = 6) had a mean circulating 11KT level about 7.5 times higher (2.61 ± 0.850 ng/ml). The 3 groups were significantly different (*F* = 6.199, df = 2, 14 *p* = 0.012), with a significant difference between non-breeding and courting (Tukey HSD, *p* = 0.019) as well as between parental and courting (Tukey HSD, *p* = 0.028) but not between non-breeding and parental (Tukey HSD, *p* = 0.998) (Fig. 1).

### Laboratory

**Figure 2:**
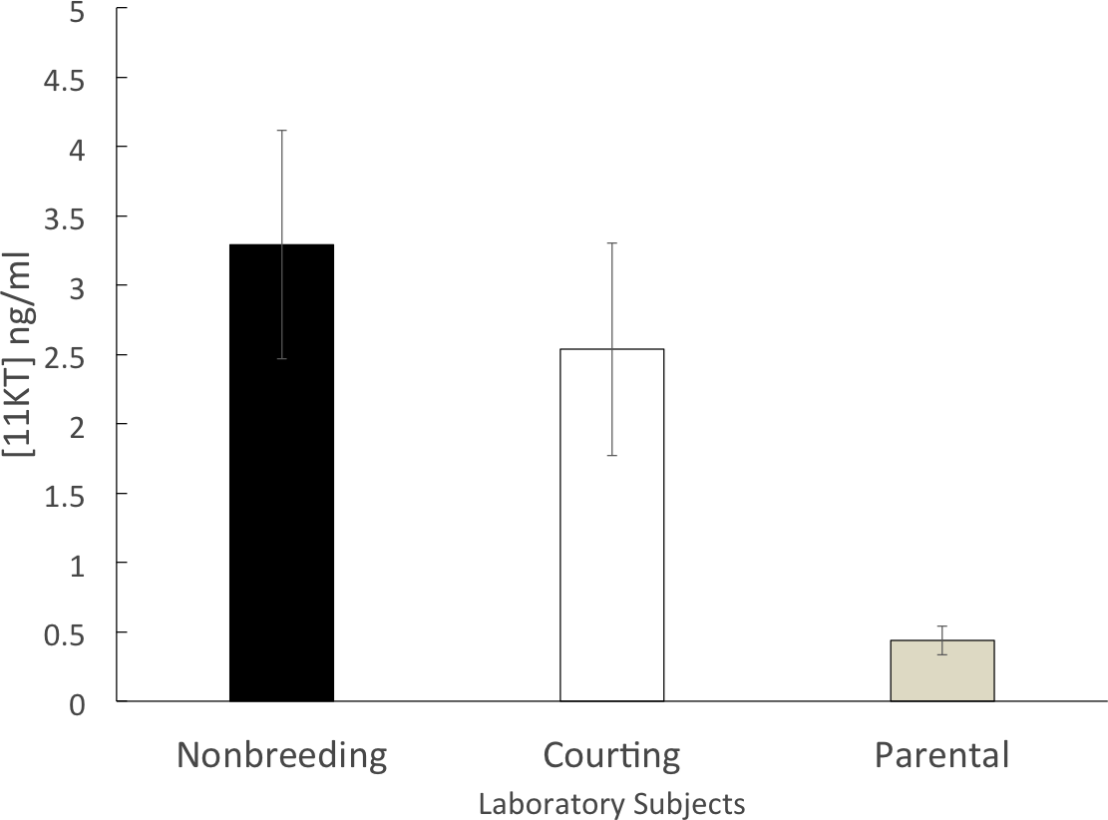
Mean (±SEM) concentration of plasma 11KT in captive (laboratory-raised) convict cichlids. Non-breeding males (black) and courting males (white) were significantly different from parental males (gray).

The parental group (*n* = 14) had a mean circulating 11KT level of 0.44 ± 0.10 ng/ml. Both the courting group (*n* = 10; 2.53 ± 0.77 ng/ml) and the non-breeding group (*n* = 11; 3.29 ± 0.82 ng/ml) had mean circulating levels of 11KT that were approximately five to six times higher than the parental group. The 3 groups were significantly different (ANOVA, *F* = 6.812, df = 2, 32 *p* = 0.003). A significant difference was found between the non-breeding and parental groups (Tukey HSD, *p* = 0.004) as well as between the courting and parental groups (Tukey HSD, *p* = 0.044) but there was not a significant difference between the non-breeding and the courting groups (Tukey HSD, *p* = 0.672) (Fig. 2).

## Discussion

The first aim of our study was to profile the circulating levels of plasma 11KT in the male convict cichlid at multiple points during the reproductive cycle. Our findings indicate that both laboratory-housed and free-living male convict cichlids exhibit a profile of 11KT during the reproductive cycle with high levels of 11KT during courtship (prior to spawning) followed by a low levels of 11KT during parental care. This observed profile is similar to other teleost species (reviewed by Borg, 1994; Oliveira et al., 2002). Previous examinations of the convict cichlid 11KT hormonal profile did not specifically look at these natural states of the reproductive cycle, and so it is difficult to directly compare them to our findings, however, many similarities are obvious. For example, Sessa, Harris & Hofmann (2013) found that males that were presented with a gravid female (though not necessarily actively courting) had higher levels of 11KT than those males that were alone (albeit, these levels were not necessarily significantly different). Similarly, O’Connell, Matthews & Hofmann (2012) found that when a male convict cichlid spawned and subsequently had their brood and mate removed, 11KT was higher than in males that were actively parenting. The conditions of our study examined naturally occurring stages of the reproductive cycle (courting vs. parenting) and were not forced experimental groupings. This provides us with a better understanding of the typical levels of circulating 11KT of male convict cichlids.

The high level of circulating 11KT during courtship in both the laboratory and field subjects again suggests that 11KT may have a function in the expression of courtship behavior. This explanation is supported by research in other species in which castration led to a decrease in pre-spawning behaviors (Salek, Sullivan & Godwin, 2001; Pall, Mayer & Borg, 2002b) which could be reinstated by treatment with 11KT (Borg, 1987). If 11KT is important for pair-bonding behaviors in convict cichlids, we would expect that blocking or removing 11KT would also decrease the expression of those behaviors. This has indeed been verified in laboratory experiments in which the anti-androgen flutamide impairs the expression of male courtship behavior in the convict cichlid (van Breukelen, 2013).

The significantly lower levels of 11KT during parental care (shown here and by O’Connell, Matthews & Hofmann, 2012), a time when convict cichlid males are highly aggressive, seem to suggest that 11KT does not have a direct role in the expression of offspring defense aggressive behavior. It will be interesting to continue to examine the role of 11KT in parental care to better determine if it is the sudden decrease in circulating 11KT that leads to the expression of parental behaviors or if it is the presence of the young that suppress 11KT for these biparental fathers.

The secondary goal of our study was to generally compare the hormonal profile of the widely used laboratory populations and that of a free-living population in Costa Rica. As expected, both populations of fish showed similar levels of 11KT during courtship and parental stages. An interesting and unanticipated result was the apparent difference between the laboratory and field in the non-breeding phase. In that laboratory, subjects had circulating levels of 11KT that resembled courting males, whereas field subjects had levels of 11KT that were similar to parental males.

This apparent difference may be due to the fact that the nonbreeding group of laboratory subjects was composed of males that had been housed in a uni-sexual group tanks for an extended period of time, as compared to the field subjects, in which the males were in free contact with both males and females. Obviously, social interactions can greatly affect the hormonal levels of individuals. It has been shown in other teleost species that males may experience an change in androgens as a result of social interactions (reviewed by Oliveira et al., 2002), territory intrusions (Hirschenhauser et al., 2004), and observing interactions (Oliveira et al., 2002). Convict cichlids in the laboratory have also specifically exhibited differences in 11KT levels depending on a “social” vs. “nonsocial” status (Sessa, Harris & Hofmann, 2013). Thus, the apparent difference between the non-breeding laboratory and field subjects may be explained by differences in social interactions. This difference is important to consider when making general comparisons between lab-housed and free-living subjects.

This continued examination of the circulating level of 11KT represents an important step towards elucidating the role of 11KT in the expression of reproductive behavior of teleosts, but also in using this convict cichlid model organism to better understand the mechanisms of behavior. Because of the vast knowledge of convict cichlid behavior and the current need for integrative studies, we reason that additional modest studies such as this will be necessary to not only describe this species but also to continue to make connections between the conditions of laboratory-housed animals and their naturally occurring field counterparts.

## Supplemental Information

10.7717/peerj.949/supp-1 Supplemental Information 1 Dataset including field and laboratory EIA results for circulating 11KT in convict cichlids

## Additional Information and Declarations

### Field Study Permissions

The following information was supplied relating to field study approvals (i.e., approving body and any reference numbers): The field experiment was conducted with approval of MINAE (permiso # 48).
